# CTLA-4-Ig internalizes CD80 in fibroblast-like synoviocytes from chronic inflammatory arthritis mouse model

**DOI:** 10.1038/s41598-022-20694-7

**Published:** 2022-09-30

**Authors:** Yoko Miura, Shyuntaro Isogai, Shinji Maeda, Satoshi Kanazawa

**Affiliations:** 1 Department of Neurodevelopmental Disorder Genetics, Nagoya City University Graduate School of Medical Sciences, 1 Kawasumi, Mizuho-cho, Mizuho-ku, Nagoya, 467-8601 Japan; 2 Department of Respiratory Medicine, Allergy and Clinical Immunology, Nagoya City University Graduate School of Medical Sciences, Nagoya, Japan

**Keywords:** Rheumatoid arthritis, Antigen-presenting cells

## Abstract

CD80 interact with CD28 and CTLA-4 on antigen-presenting cells, and function in the co-stimulatory signaling that regulates T cell activity. CTLA-4-Ig is used to treat RA by blocking co-stimulatory signaling. Chronic inflammatory arthritis was induced in D1BC mice using low-dose arthritogenic antigens and treated with CTLA-4-Ig. We performed histopathology of the joints and lymph nodes, serological examination for rheumatoid factors, and flow cytometric analysis of isolated synovial cells, including CD45^−^ FLSs and CD45^+^ synovial macrophages. CTLA-4-Ig treatment ameliorated the chronic inflammatory polyarthritis. There was a decrease in the number of infiltrating lymphoid cells in the joints as well as in the levels of RF-IgG associated with a decrease in the number of B cells in the lymph nodes; more than 15% of CD45^−^ FLSs expressed CD80, and a small number of them expressed PD-L1, indicating the presence of PD-L1/CD80 cis-heterodimers in these cells. CTLA-4-Ig internalized CD80, but not PD-L1, in isolated synovial cells. Gene ontology analysis revealed that CTLA-4-Ig internalization did not significantly alter the expression of inflammation-related genes. The therapeutic effect of CTLA-4-Ig appears to extend beyond the lymph nodes into the inflamed synovial compartment through the synergistic inactivation of T cells by the CD80 and PD-L1 axes.

## Introduction

Rheumatoid arthritis (RA) is a chronic inflammatory condition, that affects the joints and is characterized by pannus formation, and subsequent progressive bone destruction. In patients with RA, an aggressive immune response of T cells results in robust inflammation with the production of immune mediators, such as cytokines. The interaction between T-cell receptors (TCRs) on T cells, and the major histocompatibility complex class II (MHC II) molecules on antigen-presenting cells (APCs) is central to the immune response. However, T-cell activation and inactivation are regulated by the presence of co-stimulatory molecules, consisting of the B7 family proteins CD80 (B7.1) and CD86 (B7.2) on APCs, and CD28 and cytotoxic T-lymphocyte-associated antigen 4 (CTLA-4) on T cells. The binding of CD80 and/or CD86 to CD28 activates T cells^1^. Aberrant expression of CD80 in pancreatic islets causes β-cell destruction in low-dose streptozotocin-induced diabetes, because the ectopic expression of CD80 may activate T cells via CD28^2^. As a negative regulator, CTLA-4 inactivates T cells, and the lack of CTLA-4 results in fatal lymphoproliferative disorders^3^.

CTLA-4-Ig (abatacept) is an immune checkpoint inhibitor that causes immunosuppression by blocking the interaction between T cells and APCs via co-stimulatory signaling^4^. CTLA-4-Ig consists of the human CTLA-4 extracellular domain, which binds to CD80 and CD86, and is fused to the genetically modified Fc region of human IgG1. The Fc region of CTLA-4-Ig has been modified to reduce its potential to induce complement-dependent and antibody-dependent cellular cytotoxicity^5^. CTLA-4-Ig treatment has several biological functions, including the suppression of monocyte migration^6^ and the inhibition of osteoclast differentiation^7^. Persistently activated autoreactive memory B cells have been associated with the development of RA^8^. Administration of CTLA-4-Ig affects the hyperreactive memory B-cell population, resulting in a reduced production of autoantibodies in RA^9–11^. CTLA-4-Ig modulates canonical pathway-related genes, such as those expressing z-chain associated protein 70 (ZAP70), janus kinase 3 (JAK3), phospholipase C, and nuclear factor of activated T cells (NFAT) in T cells^12^. CTLA-4 is internalized by trogocytosis via CTLA-4-dependent trans-endocytosis^13^. In contrast, CTLA-4-Ig internalizes CD86 and CD80 in a dynamin-dependent manner and internalization of CTLA-4-Ig does not activate NF-kB or Akt signaling^14^. However, this process may be associated with the suppression of RA-related biomarker responses, such as rheumatoid factor-IgG (RF-IgG)^15^. Immunohistochemical analysis in a previous study revealed that CTLA-4-Ig decreased the expression of CD80 in synovial cells^16^. These immunosuppressive functions occur in secondary lymphoid tissues, such as lymph nodes, and may help to improve RA. However, the mechanisms underlying these effects in the pannus are not fully understood. In an ex vivo study using CD4^+^ T cells from the peripheral blood or the synovial fluid mononuclear cells of patients with RA, the activation of the CTLA-4 by CTLA-4-Ig promoted the production of pro-inflammatory cytokines, such as interferon-γ^17^. Recent studies have shown that the cis-programmed death-ligand 1 (PD-L1, CD274)/CD80 heterodimer present on APCs activates CD28 and overcomes negative regulation by the PD-1/PD-L1 axis^18,19^. Blockage by CTLA-4-Ig inhibits the formation of cis-PD-L1/CD80 heterodimers^20^.

Blockage of CTLA-4 by an anti-CTLA-4 antibody inhibits CD28-dependent T-cell activation, and the regulation of these negative co-stimulatory molecules has been used in radical cancer therapy^21–23^. In contrast, blockage of CTLA-4 enhances autoimmune responses in experimental allergic encephalomyelitis^24^. Further, it has been reported that anti-CTLA-4 antibody therapy causes relapse of RA^25,26^. In addition, autoantibodies against CTLA-4 have been detected in systemic autoimmune diseases, including RA^27^. Therefore, the presence of autoimmune disorders is an important risk factor for tumor immunotherapy using anti-CTLA-4 antibodies.

The D1BC transgenic mouse (D1BC: DBA/1, the B7.1 gene is transcribed from the rat collagen type II promoter and enhancer) has been established as an arthritis model expressing murine B7.1 in chondrocytes and synovial cells^28^. D1BC mice do not develop joint inflammation spontaneously, but they are more sensitive to arthritogenic antigens than conventional collagen-induced arthritis (CIA) models. Severe chronic inflammatory arthritis is induced using low doses of bovine type II collagen (bColII) in contrast to conventional methods, such as those used for the induction of CIA. Some fibroblast-like synoviocytes (FLSs) are characterized as mesenchymal cells, which tend to differentiate into osteochondrogenic lineages and express cell lineage markers such as *Col1a1*, *Runx2*, *Sox9*, and *Col10a1*^29^. These cells may be involved in aberrant bone formation. Another characteristic of D1BC mice is that their serum RF-IgG and RF-IgM levels are higher than those of DBA/1 J mice (genetic background of D1BC mice)^30^. It has been established that, as the RF concentration increases, there is a higher risk of rheumatoid arthritis-associated interstitial lung disease (RA-ILD). Previously, D1BC mice showed elevated serum surfactant protein-D levels and developed RA-ILD. It has been demonstrated that IgG secretion is promoted by CD80/CD86 signaling, which regulates immunoglobulin class switching and germinal center formation^31,32^.

In this study, we examined the effects of blocking CTLA-4 using an anti-CTLA-4 antibody, or blocking CD80 with CTLA-4-Ig in D1BC mice, and the interaction between CTLA-4-Ig and CD80 in isolated synovial cells of D1BC mice.

## Results

### CTLA-4 Ig suppresses arthritis in D1BC mice

**Figure 1:**
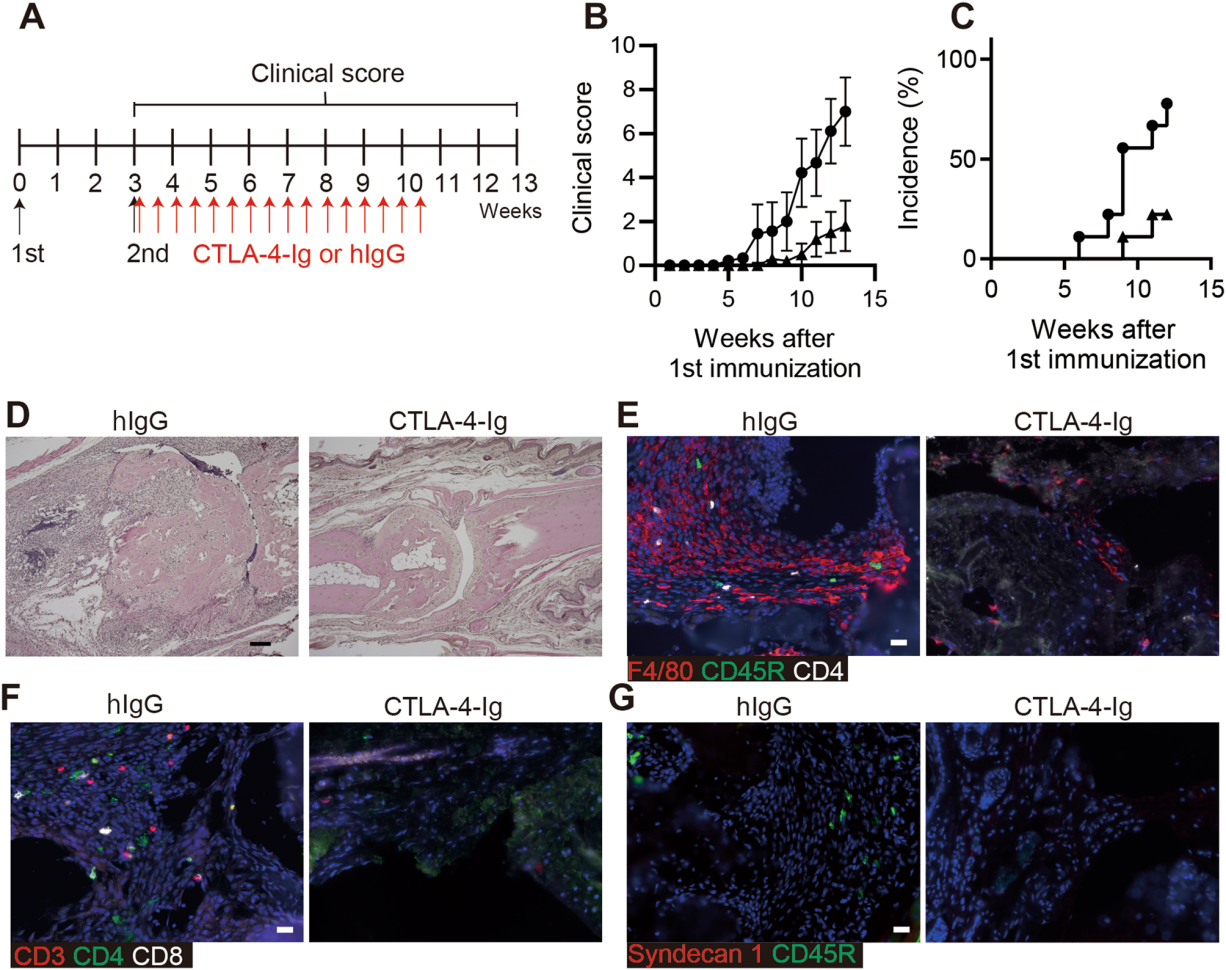
CTLA-4-Ig ameliorates chronic inflammatory arthritis in D1BC mice. (**A**) Schematic diagram indicating the timeline for the induction of chronic inflammatory arthritis with bColII, followed by the ip injection of CTLA-4-Ig or human IgG (hIgG) in D1BC mice. (**B**, **C**) The clinical score (**B**) and the percentage of incidence (**C**) of chronic inflammatory arthritis in D1BC mice treated with CTLA-4-Ig (closed triangle) and hIgG (closed circle). The clinical scores are presented as the means ± SE of ten mice. (**D**) Joints were harvested 13 weeks after the first induction and were stained by hematoxylin and eosin. (**E**–**G**) Immunohistochemical staining in the joints from CTLA-4-Ig or hIgG treated mice. Antibodies against F4/80 (red), CD45R (green), and CD4 (white) (**E**), CD3 (red), CD4 (green), and CD8 (white) (**F**), and syndecan-1 (red) and CD45R (green) (**G**) were used. Scale bars indicate 100 μm (black) and 20 μm (white).

To confirm that CTLA-4-Ig suppresses chronic inflammatory polyarthritis, D1BC mice were treated with CTLA-4-Ig or human IgG (hIgG) as a control for weeks 3–10 (two doses per week, 16 doses in total) after the first induction (Fig. 1A). CTLA-4-Ig improved arthritis in terms of clinical score and incidence, compared with hIgG (Fig. 1B, C). D1BC mice treated with CTLA-4-Ig showed less pannus formation and bone destruction, whereas the controls showed severe inflammation with synovitis (Fig. 1D).

Thereafter, the infiltrated lymphoid cells in the pannus were analyzed by immunohistochemical staining. F4/80^+^ macrophages were the major infiltrating cells, with relatively few CD45R^+^ B cells and CD3^+^ T cells. The number of infiltrating lymphocytes was dramatically reduced following CTLA-4-Ig treatment (Fig. 1E, F). The number of infiltrated plasma cells in the pannus was low and unevenly distributed (Fig. 1G and Supplementary Fig. 1).

### Administration of anti-CTLA-4 antibody exacerbates inflammatory arthritis in D1BC mice

For the suppression of CTLA-4-Ig in D1BC mice, we then determined whether the inhibition of co-stimulatory signaling exacerbates inflammatory arthritis. Anti-CTLA-4 antibody (4F10) was administered ip three times, from weeks 6 to 7 after the first induction (Supplementary Fig. 2A). Joint inflammation was observed immediately after administration of the anti-CTLA-4 antibody, suggesting that the pathological development was closer to acute inflammation than to chronic inflammation (Supplementary Fig. 2B). Anti-CTLA-4 monoclonal antibody exacerbated inflammatory arthritis and increased the incidence of disease development (Supplementary Fig. 2C).

### CTLA-4-Ig reduces the number of B cells in lymph nodes

**Figure 2:**
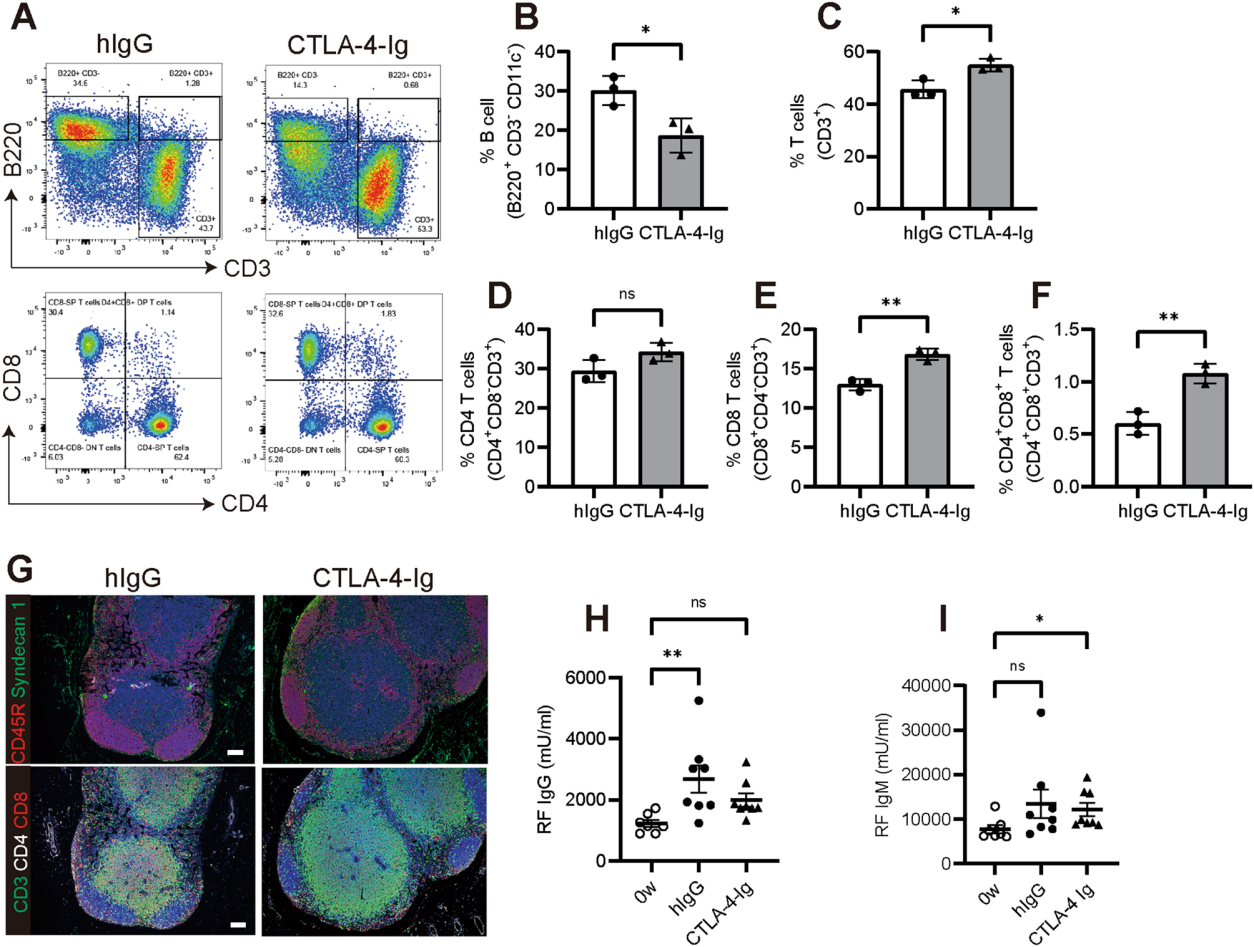
CTLA-4-Ig suppressed RF-IgG production associated with a decrease in the B-cell population. (**A**) Flow cytometric analysis of lymphoid cells from lymph nodes in CTLA-4-Ig of hIgG treated D1BC mice. Antibodies against B220, CD3, CD8, CD4, and CD11c were used. (**B**-**F**) The population of B220^+^ B cells (B), CD3^+^ T cells (**C**), CD4^+^ T cells (**D**), and CD8^+^ T cells (**E**), and CD4^+^/CD8^+^ T cells (**F**) were calculated from (**A**). Data are presented as the means ± SE of three mice in each staining. Asterisk shows *P* < 0.05, Student’s *t*. (**G**) Immunohistochemical staining of lymphocytes in lymph nodes. Antibodies against CD45R (red) and syndecan 1 (green) (upper) and CD3 (green), CD4 (white) and CD8 (red) (lower) were used. Scale bars indicate 100 μm. (**H**, **I**) Serum RF-IgG and RF-IgM levels before bColII induction (0 w) and CTLA-4-Ig or hIgG levels in the model mice (9 weeks from first induction) were measured by ELISA. Data are presented as the means ± SE of eight mice in each treatment. Asterisk indicates *P* < 0.05 (Dunnet) compared to 0 w.

We then investigated whether CTLA-4-Ig treatment altered the percentage of B220^+^ B cells (CD3^−^ and CD11c^−^) compared to that of CD3^+^ T cells in the lymph nodes. Lymphoid cells from mice treated with CTLA-4-Ig or hIgG (control) were isolated and analyzed by flow cytometry. The proportion of B220^+^ B cells decreased from 34.5% to 14.3%, whereas the number of CD3^+^ T cells increased after CTLA-4-Ig treatment (Fig. 2A-C). In these increased CD3^+^ T cells, the population of CD4^+^, CD8^+^, and double CD4^+^ and CD8^+^ cells increased compared to that in hIgG-treated mice (Fig. 2D–F). However, no histological abnormalities were observed in the overall structure and tissue distribution of the major lymphoid cells, including syndecan 1^+^ plasma B cells, in the lymph nodes with or without CTLA-4-Ig treatment (Fig. 2G).

Since high levels of serum RF are associated with the onset of RA and the reduction of the B-cell population in the lymph nodes in response to CTLA-4-Ig, we examined the serum concentrations of RF-IgG and IgM nine weeks after the first induction in comparison with the control at week 0. Administration of CTLA-4-Ig slightly decreased the serum concentration of RF-IgG, but not that of RF-IgM (Fig. 2H, I). The discontinuation of CTLA-4-Ig treatment resulted in increased serum RF-IgG levels (Supplementary Fig. 3).

### CD80 is expressed in FLSs from the pannus

**Figure 3:**
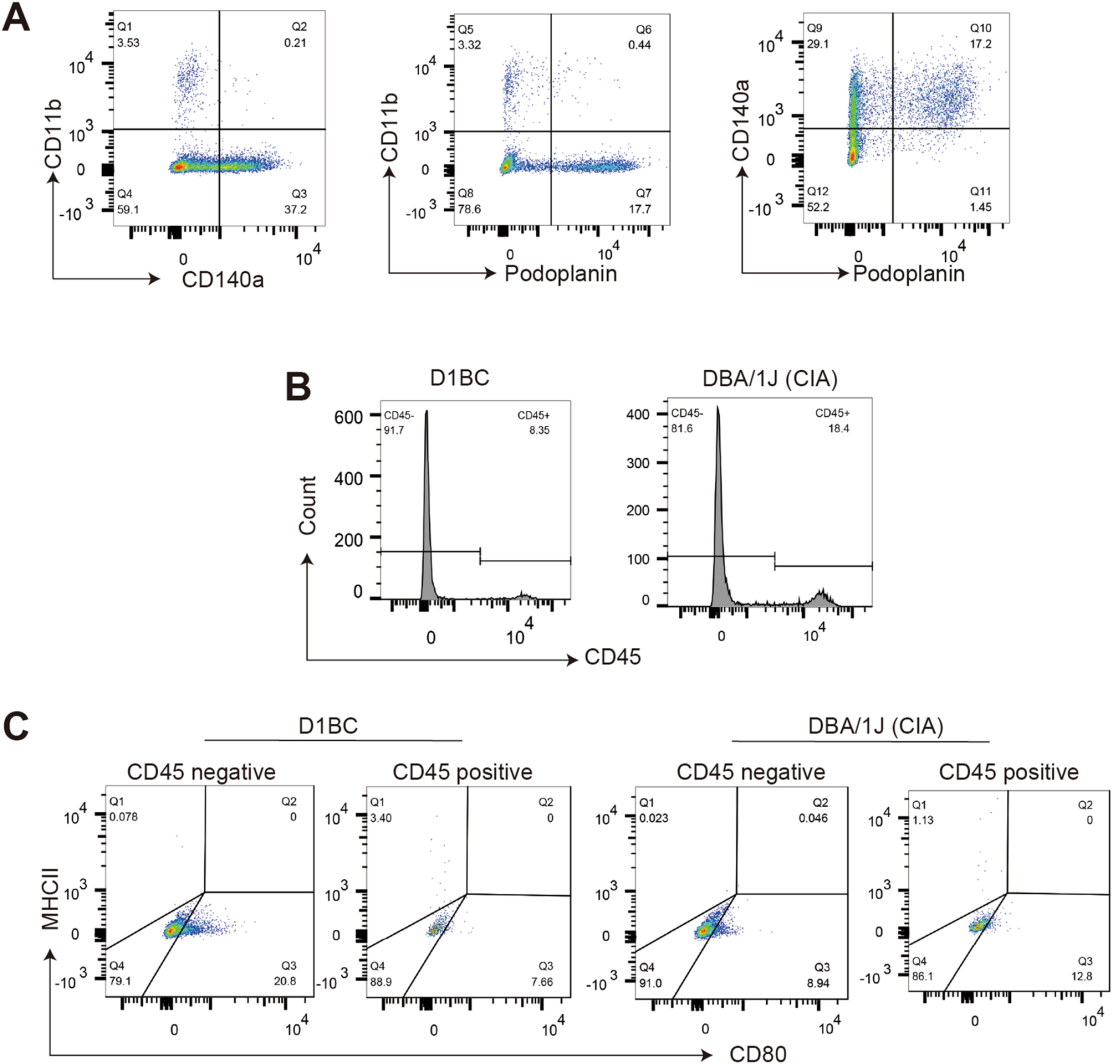
FLSs express more CD80 in D1BC mice. Synovial cells were obtained from D1BC and DBA/1 J mice (CIA) with inflammatory arthritis and cultured for one week. (**A**) Synovial cells were analyzed by flow cytometry using antibodies against CD11b, CD140a, and podoplanin. (**B**) Representative histograms of CD45 expression in D1BC and DBA/1 J mice. (**C**) MHC II and CD80 expression in CD45^+^ and CD45^−^ synovial cells. All the cells were gated as a 7-AAD-negative population.

Isolated synovial cells from the pannus of each mouse were cultured for less than one week and analyzed by flow cytometry using primary antibodies against CD11b (most macrophages, type A cells), CD140a (most fibroblasts, type B synoviocytes), and podoplanin. CD11b^+^ macrophages were observed in less than 20% of the isolated synovial cells, and few podoplanin^+^ macrophages were observed in some CD11b^+^ macrophages. Approximately 30% of podoplanin^+^/CD140a^+^ cells were defined as FLSs (Fig. 3A). We also analyzed synovial cells using primary antibodies against CD45 (CD45^+^ macrophages, CD45^−^ fibroblasts, FLSs). The population of CD45^−^ cells showed approximately 80–90% similarity with CD11b^−^ cells (Fig. 3B).

Thereafter, we analyzed the expression of CD80 and MHC II in CD45^−^ and CD45^+^ synovial cells from D1BC and DBA/1 J mice, in which joint inflammation was induced by bColII injection. Note that in DBA/1 J mice, 0.15 mg was used instead of the 0.01 mg used in D1BC mice, based on the conventional CIA model. CD80^+^ cells were detected in 16.5% and 10.3% of the CD45^−^ synovial cells in D1BC and DBA/1 J, respectively (Table 1 and Fig. 3C). In contrast, few MHC II^+^ synovial cells or CD80^+^/MHC II^+^ synovial cells were observed.

**Table 1:** Expression of MHCII and CD80 in isolated synovial cells from pannus.

	D1BC	DBA/1 J
CD45 negative	89.4 ± 4.71	78.9 ± 4.40
CD80	16.5 ± 2.58*	10.3 ± 1.05
MHC II	0.086 ± 0.0293	0.21 ± 0.107
CD80/ MHC II	0.015 ± 0.0176	0.27 ± 0.137
Double negative	83.4 ± 2.59	89.3 ± 1.21
CD45 positive	10.7 ± 4.70	21.2 ± 4.40
CD80	7.65 ± 1.70	12.2 ± 4.28
MHC II	3.12 ± 0.647*	1.15 ± 0.0720
CD80/ MH CII	N.D	0.060 ± 0.0300
Double negative	7.65 ± 1.70	12.2 ± 4.28

Data are presented as mean ± SE of each cell surface marker in isolated synovial cells (four mice). Asterisks show *P* < 0.05 compared with DBA/1 J mice. N.D. indicates not detected.

### Synovial cells of D1BC mice express both CD80 and PD-L1

Recent studies have shown that CD80 on APC heterodimerizes with PD-L1. As the cis-PD-L1/CD80 heterodimer interacts with CD28 on T cells and does not bind to PD-1, T cells are fully activated^18,19^. This suggests that the expression of both CD80 and PD-L1 in APC enhances autoimmune diseases, including RA.

**Figure 4:**
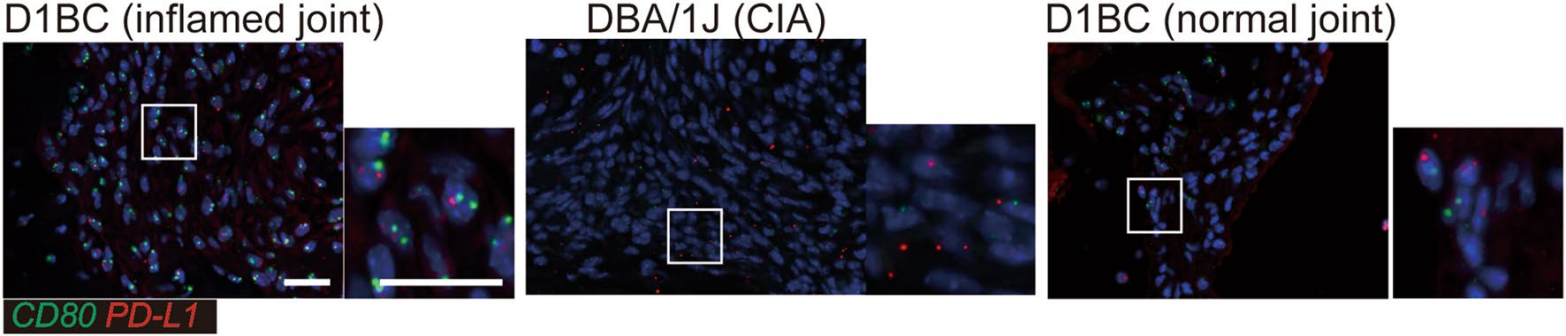
Co-expression of CD80 and PD-L1 in synovial cells. In situ hybridization of *CD80* (green) and *PD-L1* (red) in D1BC and DBA/1 J mice with chronic inflammatory arthritis and in the control (synovial membrane in D1BC mice without bColII administration). Each right panel shows a magnified image of the square on the left panel. Scale bars indicate 20 μm (white bars).

Therefore, to confirm whether CD80 and PD-L1 were expressed in the synovial cells of the pannus, in situ hybridization was performed in D1BC and DBA/1 J mice. Co-expression of CD80 and PD-L1 was observed more frequently in the inflamed pannus and normal synovial membrane of D1BC mice. They were also observed in inflamed pannus, but were in low numbers in CIA (Fig. 4).

### CTLA-4 Ig internalizes CD80, but not PD-L1, in synovial cells

**Figure 5:**
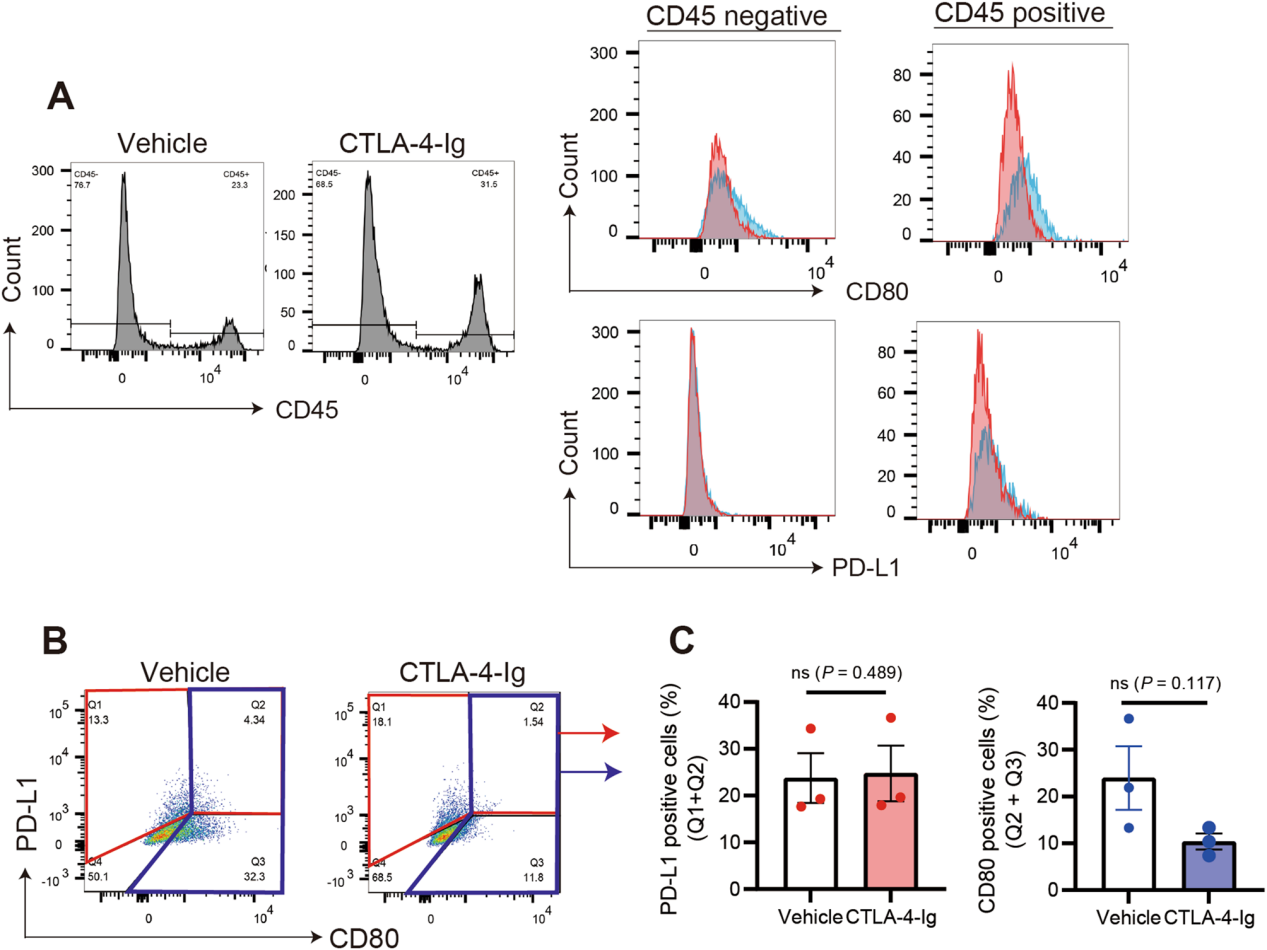
Internalization of CD80, but not PD-L1, in FLSs by CTLA-4-Ig. Internalization of CD80, but not PD-L1, by CTLA-4-Ig in CD45^+^ synovial macrophages or CD45^−^ FLSs in D1BC mice (**A**) Synovial cells were isolated from the pannus of D1BC mice with chronic inflammatory arthritis. Cells were treated with CTLA-4-Ig for 2 h following treatment with a dynamin inhibitor, and analyzed by flow cytometry using antibodies against CD45, CD80, and PD-L1. Each histogram represents CD80 or PD-L1 expression in CTLA-4-Ig-treated (red) and untreated cells (blue). (**B**) Flow cytometric analysis showing CD80 and PD-L1 expression in CTLA-4-Ig-treated and untreated cells. (**C**) CD80^+^ (Q2 + Q3) and PD-L1^+^ (Q1 + Q2) cells were compared between the CTLA-4-Ig-treated and untreated synovial cells. Data are presented as the means ± SE of three mice in each treatment. ns, non-significant, Student’s *t*.

We investigated whether CTLA-4-Ig bound to CD80 and was internalized via endocytosis. It was difficult to detect the internalization of CTLA-4-Ig-bound CD80 without inhibiting clathrin-mediated endocytosis using flow cytometry (Supplementary Fig. 4). However, binding of CTLA-4-Ig to CD80 did not prevent the recognition of CD80 by anti-CD80 antibodies in isolated synovial cells. Therefore, we examined CTLA-4-Ig internalization by CD80 using a dynamin inhibitor, which blocks the final scission step of endocytosis^33^. CD80 molecules were internalized by CTLA-4-Ig treatment in both CD45^+^ and CD45^−^ synovial cells (Fig. 5A and Supplementary Fig. 5). The dynamin inhibitor internalized CTLA-4-Ig bound to CD80, resulting in loss of CD80 molecules from the cell surface. Although a small number of CD80^+^/PD-L1^+^ cells were observed in the histological analysis (Fig. 4), they were detected in both CD45^+^ and CD45^−^ cells in the flow cytometric analysis (Fig. 5B). There was no change in PD-L1 expression after CTLA-4-Ig treatment of CD45^+^ and CD45^−^ synovial cells (Fig. 5A). The population of CD80^+^ cells (Q2 and Q3 in Fig. 5C) increased. In contrast, PD-L1^+^ cells (Q1 and Q2 in Fig. 5C) did not change, suggesting that CTLA-4-Ig specifically internalized CD80 but did not internalize PD-L1 simultaneously (Fig. 5C).

Thereafter, we analyzed the effect of internalized CTLA-4-Ig on synovial cells by microarray gene expression. There were no significant differences in matrix metalloproteases, interleukins, chemokines, or other cytokines (Supplementary Fig. 6). Significant alterations in the expression of *Mmp3*, *Mmp8*, *Mmp9*, *Mmp10*, *Mmp12*, *Il6*, *Ifng*, and *Ifna2* in synovial cells upon CTLA-4-Ig treatment were not observed when analyzed by qPCR (Supplementary Fig. 7, The △CT for *Ifng* and *Ifna2* were below detectable levels, so data for both are not shown*.*).

## Discussion

CTLA-4-Ig regulates the immune activation of the CD28-CD80/86 axis and has been used to treat RA. We previously established a mouse model of chronic inflammatory arthritis that expresses CD80 in synovial cells and chondrocytes of the joints. Administration of CTLA-4-Ig suppressed chronic inflammatory arthritis and decreased serum RF-IgG levels in D1BC mice, whereas exacerbation of RA was observed with anti-CTLA-4 antibody treatment. Under these circumstances, the anti-CTLA-4 antibody may function predominantly in the inflamed joint compartment. CTLA-4-Ig internalized CD80, but not PD-L1, in CD45^−^ FLSs in addition to CD45^+^ macrophages. It has been suggested that CTLA-4-Ig suppresses the activation of T cells in the joint cavity and lymph nodes.

CTLA-4-Ig affects plasmablasts during B-cell development and reduces the production of serum IgG and autoantibodies, such as RF-IgG and anti-citrullinated protein antibody (ACPA)^14,15^. In D1BC mice, there was a decrease in the number of infiltrating plasma cells in the joints, and B cells in the lymph nodes following CTLA-4-Ig treatment (Fig. 1G, 2B, and supplementary Fig. 1). The decrease in serum RF-IgG levels may reflect these changes (Fig. 2H). We did not measure mouse ACPA. This was because of the difficulty in measuring appropriate concentrations due to cross-reactivity of mouse ACPA with non-citrulline antigens^34^. RF, and to a lesser extent ACPA, are also risk factors of RA-ILD^35,36^. Therefore, the reduction in serum RF levels by CTLA-4-Ig treatment may have additional therapeutic effects on the extra-articular manifestations of RA. Since D1BC mice develop RA-ILD, it would be interesting to determine whether CTLA-4-Ig administration can prevent RA-ILD in addition to RA in D1BC mice. Serum RF levels increased with disease progression, and higher serum RF levels were detected in D1BC mice that were not injected with an arthritogenic antigen than in DBA/1 J mice^30^.

Macrophage-like (CD45^+^) synovial cells and CD45^−^ FLSs are the major cell populations in the lining and sub-lining layers of the pannus. Synovial macrophages express co-stimulatory signaling molecules, such as CD80 and PD-L1, which are involved in joint inflammation. We demonstrated that a limited number of FLSs expressed CD80 in the CIA model using DBA/1 J and D1BC mice (Fig. 3B). Interestingly, we found that some synovial cells expressed PD-L1 (Figs. 4 and 5B). In the histogram in Fig. 5, more CD80-positive cells were observed than PD-L1-positive cells, and both showed similar fluorescence intensities. Therefore, PD-L1 molecules may form heterodimers with CD80 in most CD80 and PD-L1 double-positive cells^18^. In contrast, CTLA-4-Ig treatment disrupts the formation of cis-PD-L1/CD80 heterodimers, allowing more CD80 homodimers to form^37^. However, CTLA-4-Ig bound CD80 molecules were internalized in isolated synovial cells. CTLA-4-Ig blocks the CD28/CD80 axis, whereas the PD-1/PD-L1 axis remained unaffected, which may have more effectively suppressed the activated T cells. In this context, the combination of CTLA-4-Ig treatment with agonistic PD-1 antibodies and others may be more effective in the treatment of RA. Moreover, *CD80* and *PD-L1* were co-expressed in the synovial cells of D1BC mice, even before disease onset. This suggests that CD28-mediated T-cell activation in the joint compartment of the D1BC mouse model exacerbates chronic inflammatory arthritis. The interaction of T cells with isolated primary FLSs treated with CTLA-4-Ig requires further investigation; however, only a limited number of primary synovial cells can be obtained from the CIA and D1BC mouse models. Future studies should combine the analysis of cell–cell interactions between CTLA-4-Ig-treated primary synovial cells and T cells with single-cell RNA-seq (scRNA-seq) analysis, which will reveal more details regarding CTLA-4-Ig-induced immune suppression in joints^38^. One caveat of our data is that we could not provide direct evidence of internalized CD80 and CTLA-4-Ig molecules in the synovial cells after CTLA-4-Ig treatment owing to the limited number of synovial cells that can be isolated from the pannus of D1BC mice.

In a previous study, CTLA-4-Ig treatment regulated the expression of several genes but had no significant effect on B-cell populations in the peripheral blood^39,40^. The effects of pro-inflammatory cytokines, such as IL-6 and TNF-α on synoviocytes, are controversial^11,16^. Internalization of CTLA-4-Ig did not affect gene expression in synovial cells, suggesting that CTLA-4-Ig directly regulates T cells and indirectly affects B-cell development. In general, various studies have reported the role of CD80/86 in T-cell activation, but little is known about the expression levels of CD80/86 and their function in synoviocytes, especially in FLSs, of the joint compartments^41^. We were interested in finding evidence of CD80-expressing synovial cells in arthritis models, such as the CIA and D1BC mouse models. In particular, CD80 expression was enhanced in D1BC mice because the CD80 gene was introduced under the control of the type II collagen promoter and enhancer. Direct imaging of labeled CTLA-4-Ig bound to infiltrated macrophages and fibroblasts in inflamed joints has been reported^42^. CTLA-4-Ig bound to Lin^−^, CD45^−^, CD140a^+^, and CD31^−^ fibroblast cells in the inflamed synovium; these fibroblasts expressed CD80, but not CD86 or I-A/I-E. In histological analysis, FLSs were found to comprise at least three types of cells in terms of their osteochondrogenic lineage, including *Sox9*-, *Runx2*-, and *Col10a1*-positive cells; FLSs thus show cellular heterogeneity^28^. Moreover, four fibroblast subpopulations from synovial tissue, and eight putative clusters of CD45^−^ and podoplanin^+^ cells have been identified^43^. In synovial cells isolated from D1BC mice, the CD140a^+^ and podoplanin^+^ cell populations comprised 17.2%, which indicates a greater complexity of these synovial cells than expected of the synovial cells isolated from an inflamed synovium. Therefore, further investigation of the heterogeneity of CD45^−^ and CD80^+^ FLSs using different surface markers is needed. In summary, even a small number of FLSs in the pannus express both CD80 and PD-L1 and may regulate T-cell activation in the joints. This induces chronic aggregation of immune chain reactions in patients with RA. CTLA-4-Ig inhibits the interaction between FLSs expressing PD-L1/CD80 and T cells.

In conclusion, we showed that CTLA-4-Ig treatment ameliorated chronic inflammatory polyarthritis in a mouse model by reducing the production of serum RF-IgG associated with a decrease in the number of B cells in the lymph nodes. In situ hybridization suggested that cis-heterodimers of PD-L1 and CD80 are present in synovial cells isolated from the inflamed synovium. Moreover, we found that CTLA-4-Ig internalized CD80, but not PD-L1, in CD45^−^ FLSs and CD45^+^ synovial macrophages. Histochemical analysis revealed immunosuppression by CTLA-4-Ig in the joint compartment; further experiments in vitro with isolated CD80^+^ FLSs are needed to confirm T-cell inactivation by CTLA-4-Ig. However, we concluded that CTLA-4-Ig exerts immunosuppression in the inflamed synovial compartment through two-way cooperation between the CD80 and PD-L1 axes.

## Methods

### Mouse model of chronic inflammatory polyarthritis

D1BC mice express murine B7.1 in their chondrocytes and synovial membranes^28^. A low dose of bColII (10 μg per mouse) was used to induce chronic inflammatory arthritis in D1BC mice, and a dose of 150 μg per mouse was used for conventional CIA in DBA/1 J mice^44^. Briefly, 7–8-weeks-old D1BC or DBA/1 J mice were housed in a pathogen-free animal care facility at Nagoya City University Medical School in accordance with institutional guidelines. Mice were anesthetized with isoflurane and administered bColII emulsified with an equal volume of complete Freund’s adjuvant (BD Biosciences, Sparks, MD, USA). The same protocol was used for secondary induction with bColII, except for the use of incomplete Freund’s adjuvant (BD Biosciences).

### Anti-CTLA-4 antibody treatment

B-cell hybridoma for anti-CTLA-4 monoclonal antibody (clone UC10-4F10-11, Hamster IgG) was provided by Dr. J. Bluestone from the University of California, San Francisco. B-cell hybridoma was cultured in DMEM (Sigma-Aldrich, Steinheim, Germany) with 10% fetal bovine serum (Gibco, Life Technologies, Carlsbad, CA, USA), 0.15% sodium bicarbonate (Gibco, Life Technologies), 0.01 mM non-essential amino acids (Gibco, Life Technologies), 0.05 mM 2-mercaptethanol (Fujifilm-Wako, Tokyo, Japan), and 500 U/ml penicillin–streptomycin (Gibco, Life Technologies). The monoclonal antibody was purified using a Protein A Sepharose 4 Fast Flow system (GE Healthcare, Uppsala, Sweden). Anti-CTLA-4 antibodies (0.1 mg) were injected intraperitoneally (ip) three times at 6 weeks in RA-induced D1BC mice. The same amount of saline was injected as the negative control.

### CTLA-4 Ig treatment

CTLA-4-Ig (abatacept,100 μg/ 0.1 ml/ mouse, Bristol-Myers Squibb, Princeton, NJ, USA) and human IgG (hIgG, 100 μg/ 0.1 ml/mouse, supplied by Pharma Foods International, Kyoto, Japan) as a control, were injected ip two times a week, from weeks 3 to 10 after the first induction (Fig. 1A).

### ELISA

Serum RF-IgG and RF-IgM concentrations were measured using ELISA, according to the manufacturer’s instructions (Fujifilm-Wako-Shibayagi, Gunma, Japan). Serum obtained before bColII induction was used as the control.

### Histopathology

Each joint and lymph node was fixed in 4% paraformaldehyde/PBS, decalcified in 2.5% EDTA, and stored in 7% sucrose at 4 °C for one month. Paraffin Sects. (2 μm) were stained with hematoxylin and eosin (H&E; Muto, Tokyo, Japan) for immunohistochemical analysis. The sections were stained with the following primary antibodies: rat anti-F4/80 (Bio-Rad), rabbit anti-CD3 (Genemed Biotechnologies, South San Francisco, CA, USA), rat anti-PTPRC/CD45R (Aviva Systems Biology, San Diego, CA, USA), rabbit anti-syndecan 1 (Bioss Antibodies, Woburn, MA, USA), anti-CD4, and anti-CD8 (Cell Signaling Technology, Danvers, MA, USA). Histofine simple stain mouse MAX-PO secondary antibodies and the Opal multiplex fluorescent immunohistochemistry system (Akoya Biosciences, Marlborough, MA, USA) were used according to the manufacturers’ protocols. All images were captured using a fluorescence microscope (BZ-X710; Keyence, Osaka, Japan).

### Flow cytometric analysis of mouse lymph nodes

Cells were separated by density gradient centrifugation using Ficoll-Paque Plus (GE Healthcare) and resuspended in flow cytometry buffer (Hanks’ Balanced Salt Solution supplemented with 2% heat-inactivated fetal calf serum, 0.05% sodium azide, and 0.5% EDTA). The cells were stained with the following fluorochrome-labeled monoclonal antibodies: anti-CD3-PE-Cyamine7 (Clone 17A2, Invitrogen, Waltham, MA, USA), anti-CD4-FITC (Clone RM4-5, Invitrogen), anti-CD8-eFluor 450 (Clone 53–6.7, Invitrogen), anti CD11c-PE (Clone N418, BD Biosciences), and anti-CD45R(B220)-eFluor780 (Clone RA3-6B2, Biolegend, Hercules, USA), and incubated at 4 °C for 30 min in the dark. These cells were then analyzed using a FACSCanto II (BD Biosciences).

### In situ* hybridization*

In situ hybridization for *CD80* and *PD-L1* (*CD274*) was performed using the RNAscope Multiplex Fluorescent Reagent Kit v2 (Advanced Cell Diagnostics, Newark, CA, USA), according to the manufacturer’s instructions.

### Synovial cell isolation and flow cytometry analysis of the cells

Synovial cells were isolated from the pannus and incubated in dissection buffer, Hanks’ Balanced Salt Solution (HBSS, Gibco) supplemented with 48.1 μg/ml DNase I (Worthington Biochemicals, Lakewood, NJ, USA), 1.92 mg/ml Collagenase type III (Worthington Biochemicals), and 1.77 U/ml Dispase (Gibco) at 37 °C for 30 min. After centrifugation at 100 × *g* for 3 min, the cells were resuspended in DMEM with 10% FBS supplemented with penicillin–streptomycin, and cultured at 37 °C in 5% CO_2_ for five days. For flow cytometry analysis, approximately 1 × 10^5^ cells of isolated fibroblast-like synoviocytes (FLSs) blocked FCγR with anti CD16/CD32 antibody (BD Biosciences) at 4 °C for 5 min. After washing with PBS containing 2% FBS, the cells were analyzed using the following primary antibodies: CD11b-PE (Clone M1/70, BD Biosciences), podoplanin-BV421 (Clone 8.1.1, BioLegend), Pdgfra (CD140a)-APCvio770 (Clone REA637, Miltenyi Biotec, NRW, Germany), CD45-APC (Clone, 30-F11, BioLegend), MHC class II-PE (Clone REA813, Miltenyi Biotec), CD80-BV421 (Clone 16-10A1, BD biosciences), CD274 (PD-L1)-PE (Clone 10F.9G2, BioLegend), and 7-Aminoactinocynin D (7-AAD) (BD Biosciences). These cells were analyzed using a FACSCanto II (BD Biosciences). To block CD80 internalization by the endocytosis inhibitor, the synovial cells were pre-incubated with 80 μM Dynasore hydrate (Sigma-Aldrich) in DMEM without FBS for 30 min, and then CTLA-4 Ig (1,000 ng/ml) was added and incubated at 37 °C for 2 h. This was followed by flow cytometry analysis as described above.

### RNA microarray and quantitative PCR of synovial cells

Isolated synovial cells (approximately 1 × 10^5^ cells) were cultured in DMEM supplemented with 10% FBS overnight at 37 °C in a 5% CO_2_ atmosphere. Thereafter, abatacept (100 μg/ml), human IgG (100 μg/ml), and a vehicle (DMEM with 10% FBS as a control) were added to the medium and the cells were incubated at 37 °C, under 5% CO_2_ for 48 h. Total RNA extracted from the synovial cells was used as the sample in the ReliaPrep RNA Tissue miniprep system (Promega, Madison, WI, USA). For RNA array, Toray’s 3D-Gene Mouse Oligo chip 24 k (Toray Industries, Tokyo, Japan) was used for RNA expression profiling according to the manufacturer’s instructions. Briefly, for efficient hybridization, this microarray adopted a columnar structure to stabilize spot morphology and enable microbead agitation. Total RNA was labeled with Cy5 using the Amino Allyl Message AMP II amplified RNA (aRNA) Amplification Kit (Applied Biosystems, Waltham, MA, USA). Cy5-labeled aRNA pools were mixed with hybridization buffer and hybridized for 16 h. Hybridization was performed according to the manufacturer’s instructions (www.3d-gene.com). Hybridization signals were obtained using a 3D-Gene Scanner (Toray Industries) and processed using 3D-Gene Extraction software (Toray Industries). The detected signals for each gene were normalized using the global normalization method (the median of the detected signal intensity was adjusted to 25). Gene expression data have been archived as Minimum Information About a Microarray Experiment (MIAME) and are publicly available as record GSE200403 in the NCBI’s Gene Expression Omnibus database. For quantitative PCR (qPCR), cDNA was synthesized using ReverTra Ace qPCR RT Master Mix with gDNA Remover (TOYOBO, Osaka, Japan). Quantitative polymerase chain reaction (qPCR) was performed using the PrimeTime Gene Expression Master Mix (Integrated DNA Technologies, Coralville, IA, USA). The relative expression of each gene was determined by an internal control using Hprt for each sample.

### Statistical analyses

Data are expressed as the mean ± standard error (SE). Differences between CTLA-4-Ig and human IgG were evaluated using Student’s *t*-test for clinical scores, lymphoid cell populations, and qPCR. Serum RF-IgG and RF-IgM levels were measured and evaluated using one-way analysis of variance (ANOVA), followed by Dunnett’s test for parametric data and the Kruskal–Wallis test followed by Dunn’s test for nonparametric data (GraphPad Prism 9; GraphPad Software, Inc., La Jolla, CA, USA). Statistical significance was set at *P* < 0.05.

### Ethics approval

All mouse experiments were performed according to the rules and regulations of the Fundamental Guidelines for Proper Conduct of Animal Experiments and Related Activities in Academic Research Institutions under the jurisdiction of the Ministry of Education, Culture, Sports, Science, and Technology of Japan, and in accordance with ARRIVE guidelines, and approved by the Committee on the Ethics of Animal Experiments of Nagoya City University.

## Supplementary Information

Supplementary Information.

## Data Availability

Gene expression data have been archived as Minimum Information About a Microarray Experiment (MIAME) and are publicly available as records GSE200403 in NCBI’s Gene Expression Omnibus database.
