# Supplementary material for: CTLA-4-Ig internalizes CD80 in fibroblast-like synoviocytes from chronic inflammatory arthritis mouse model
Source: Sci Rep. 2022 Sep 30;12:16363. doi: 10.1038/s41598-022-20694-7 (PMC9525600; doi:10.1038/s41598-022-20694-7)
Supplement: Supplementary file 1 — Supplementary Information. [file 41598_2022_20694_MOESM1_ESM.pdf]

CTLA-4-Ig internalizes CD80 in fibroblast-like synoviocytes from chronic inflammatory arthritis mouse model

Yoko Miura[1)], Shyuntaro Isogai[2)], Shinji Maeda[2)], and Satoshi Kanazawa[1) *].

Supplementary Information

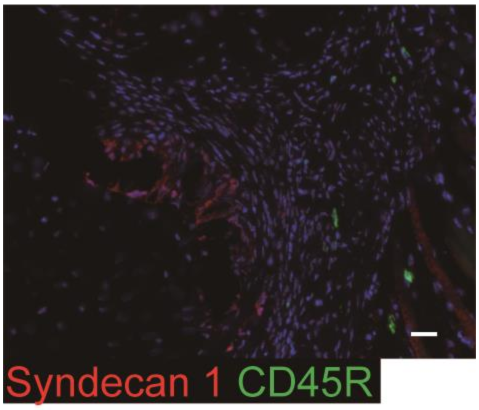

Supplementary Figure 1. Clusters of Syndecan-1^{+} plasma cells in pannus. Immunohistochemical staining of syndecan-1 (red) and CD45R (green) in hIgG-treated D1BC mice Scale bars indicate 20 μm.

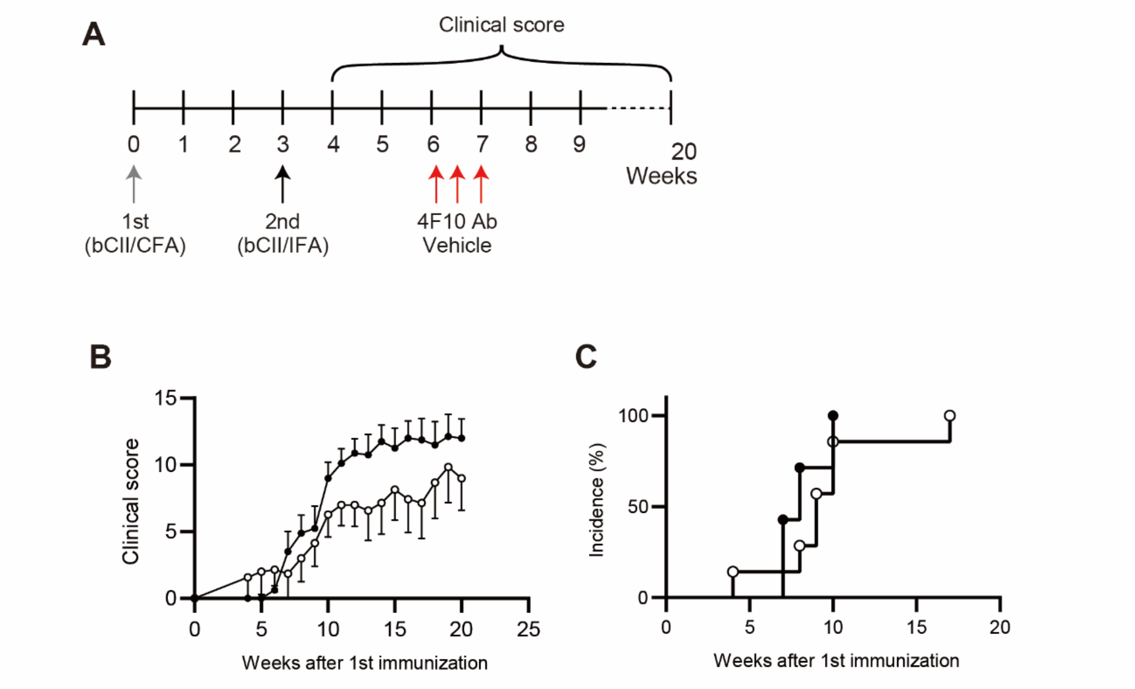

Supplementary Figure 2. Anti-CTLA-4 antibody induced acute and severe inflammatory arthritis in D1BC mice.

(A) Schematic diagram indicating the timeline of D1BC mice induced with bColII, followed by ip injection of anti-CTLA-4 antibody (4F10) or vehicle. (b-c) Clinical score (B) and incidence (C) of inflammatory arthritis treated with 4F10 antibody (closed circles) and vehicle (PBS, open circles) were assessed. Clinical score data are presented as the mean ± SE of eight mice.

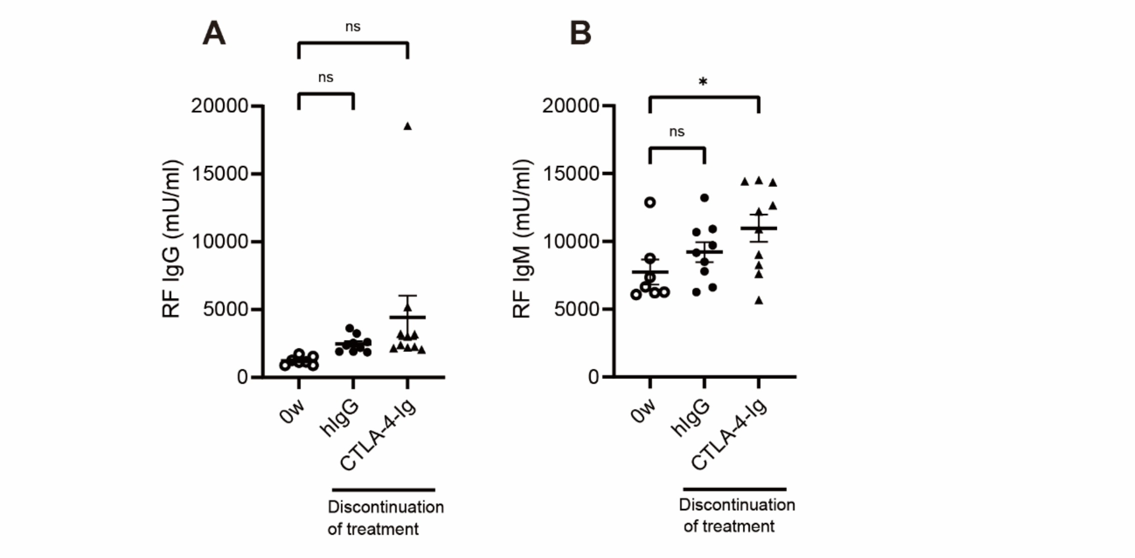

Supplementary Figure 3. Serum RF-IgG levels increased after discontinuation of CTLA-4-Ig treatment.

(A) Serum RF-IgG and (B) IgM levels were measured by ELISA. Sera from CTLA-4-Ig and hIgG-treated D1BC mice were collected three weeks after treatment discontinuation, except for the controls (0 w). Data are presented as the mean ± SE of eight mice in each group. Asterisks indicate $P < 0.05$, Dunnett's test, compared with 0 w.

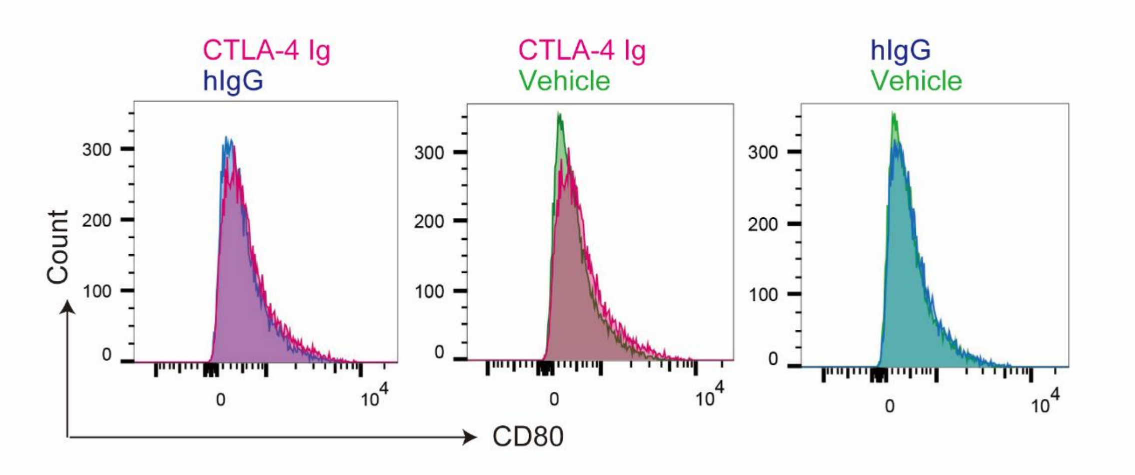

Supplementary Figure 4. CTLA-4-Ig binding does not prevent recognition of CD80 by anti-CD80 antibody in isolated synovial cells.

Synovial cells were isolated from the pannus of D1BC mice with chronic inflammatory arthritis. Cells were treated with CTLA-4-Ig (100 μg/ml), hIgG (100 μg/ml), or vehicle for 2 h and then analyzed by flow cytometry using anti-CD80 antibody. Histograms for CD80 represent CTLA-4-Ig (red) and hIgG (blue), CTLA-4-Ig (red) and vehicle (green), or hIgG (blue) and vehicle (green) overwrite data, respectively.

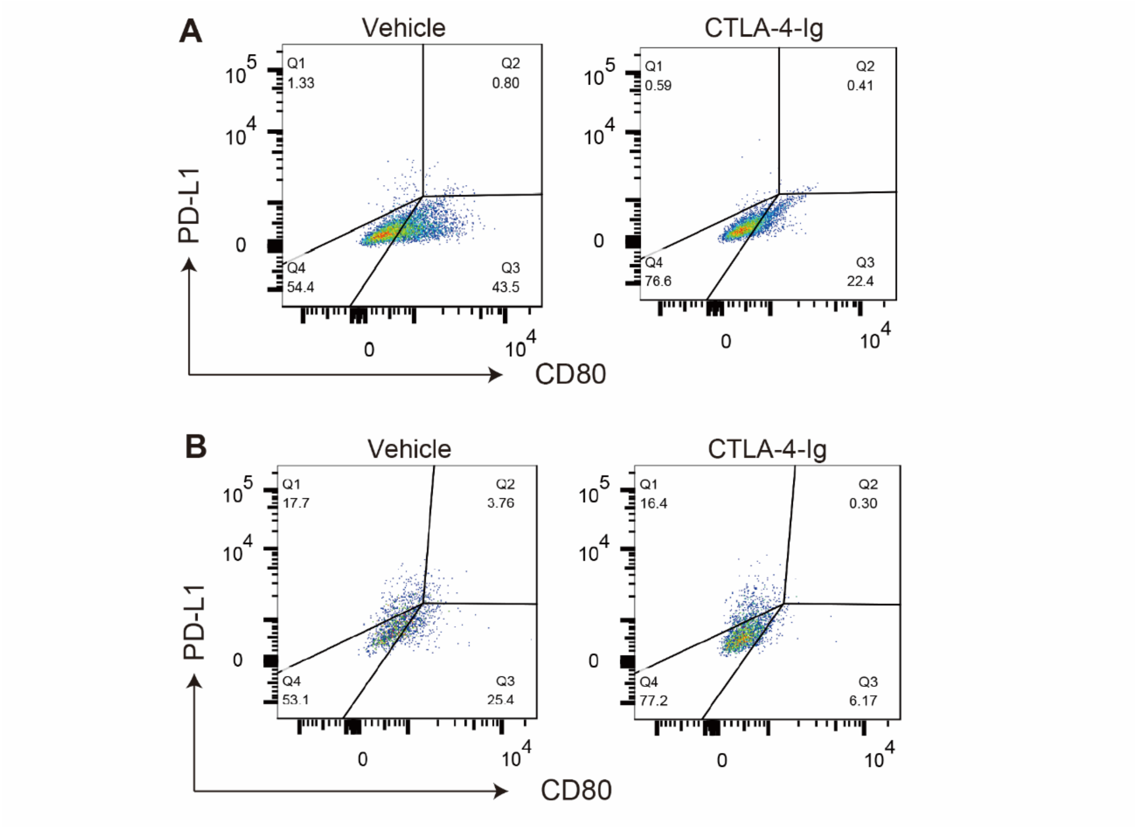

Supplementary Figure 5. CD80 molecules in CD45⁻ FLSs were internalized by CTLA-4-Ig treatment but not in PD-L1.

Internalization of CD80 molecules after CLTA-4-Ig treatment was observed in both $CD45^+$ and $CD45^-$ cells. Flow cytometric analysis of CD80 and PD-L1 expression in $CD45^-$ FLSs (A) and $CD45^+$ macrophages (B) in the pannus of D1BC mice. Isolated synovial cells were treated with or without CTLA-4-Ig for 2 h following treatment with dynamin inhibitor and analyzed by flow cytometry using anti-CD45, CD80, and PD-L1 antibodies.

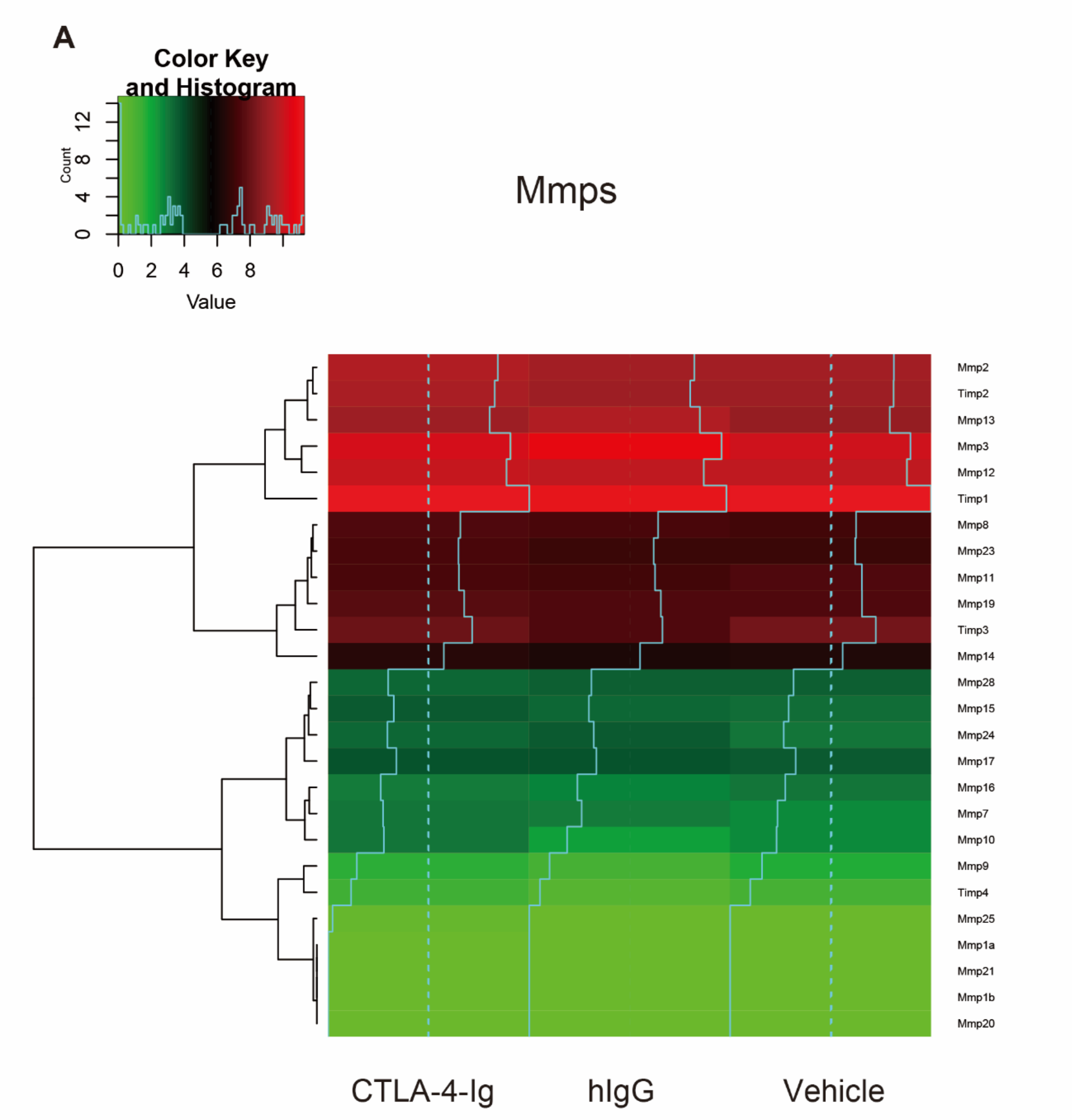

Supplementary figure 6A. No significant mRNA alternation associated with CTLA-4-Ig treatment in synovial cells

Microarray analysis of the isolated synovial cells treated with either CTLA-4 Ig, hIgG, or vehicle. Diagram showing heatmaps of representative metalloproteases mRNAs.

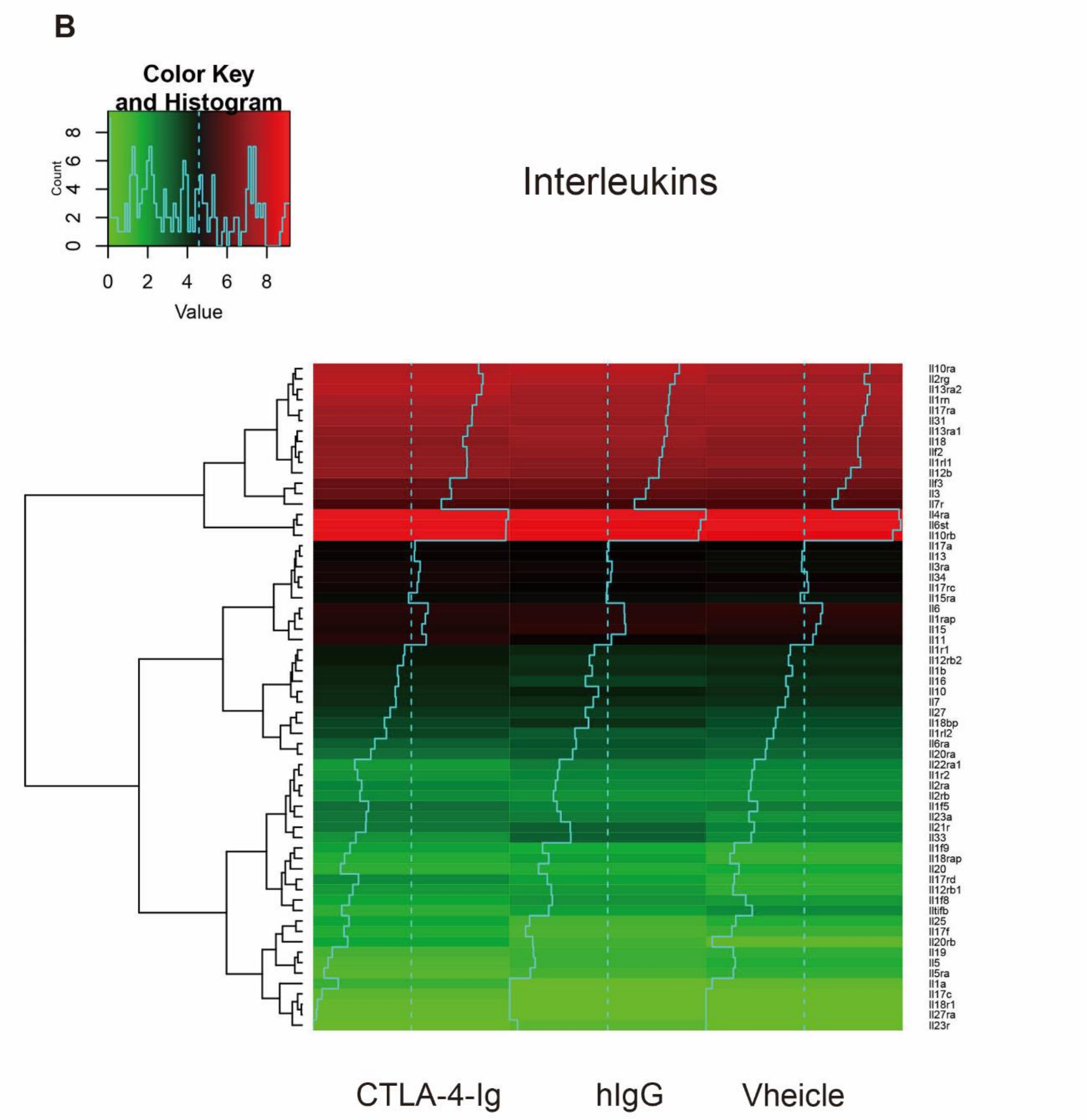

Supplementary figure 6B. No significant mRNA alternation associated with CTLA-4-Ig treatment in synovial cells

Microarray analysis of the isolated synovial cells treated with either CTLA-4 Ig, hIgG, or vehicle. Diagram showing heatmaps of representative interleukins mRNAs.

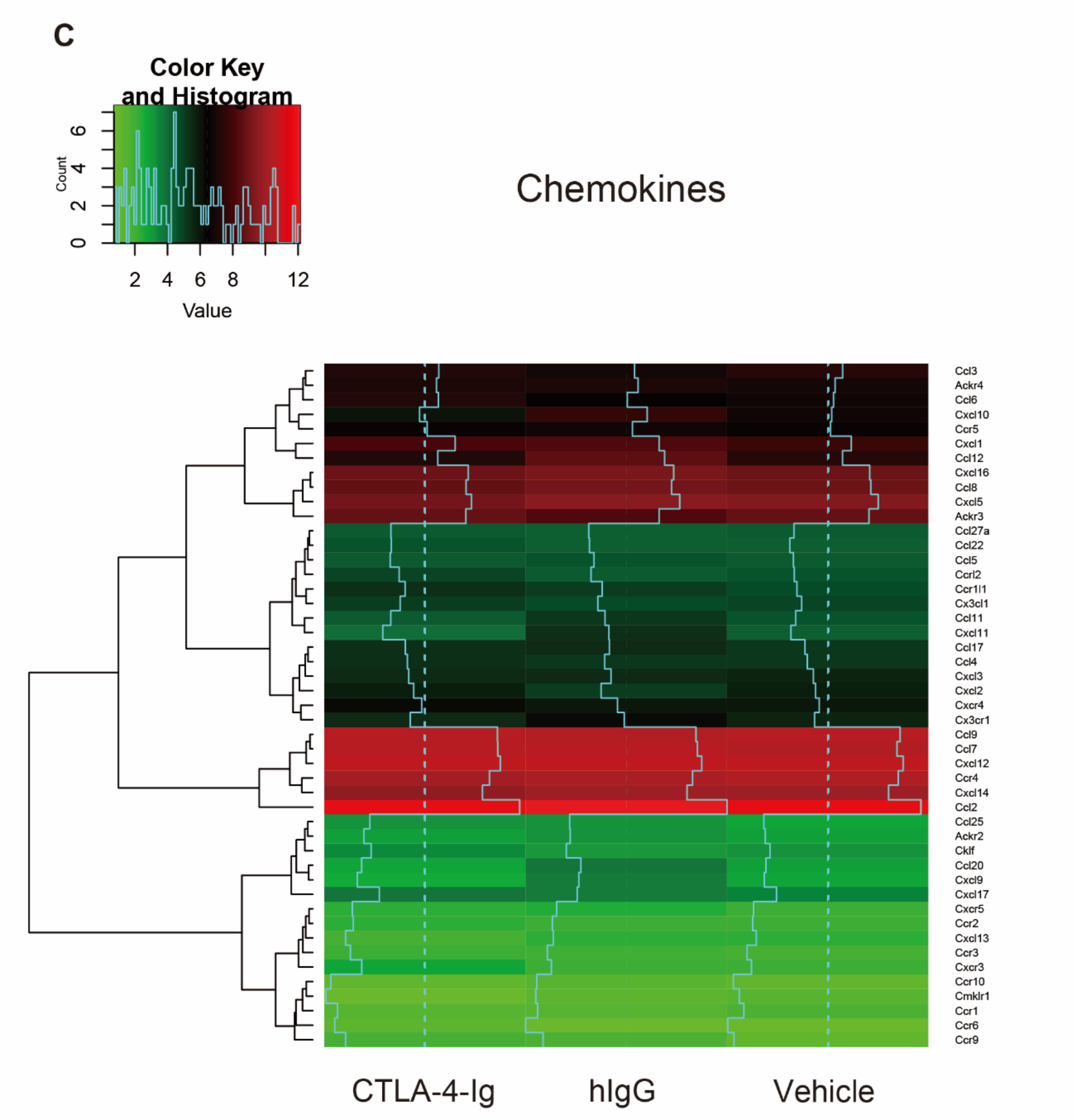

Supplementary figure 6C. No significant mRNA alternation associated with CTLA-4-Ig treatment in synovial cells

Microarray analysis of the isolated synovial cells treated with either CTLA-4 Ig, hIgG, or vehicle. Diagram showing heatmaps of representative chemokines mRNAs.

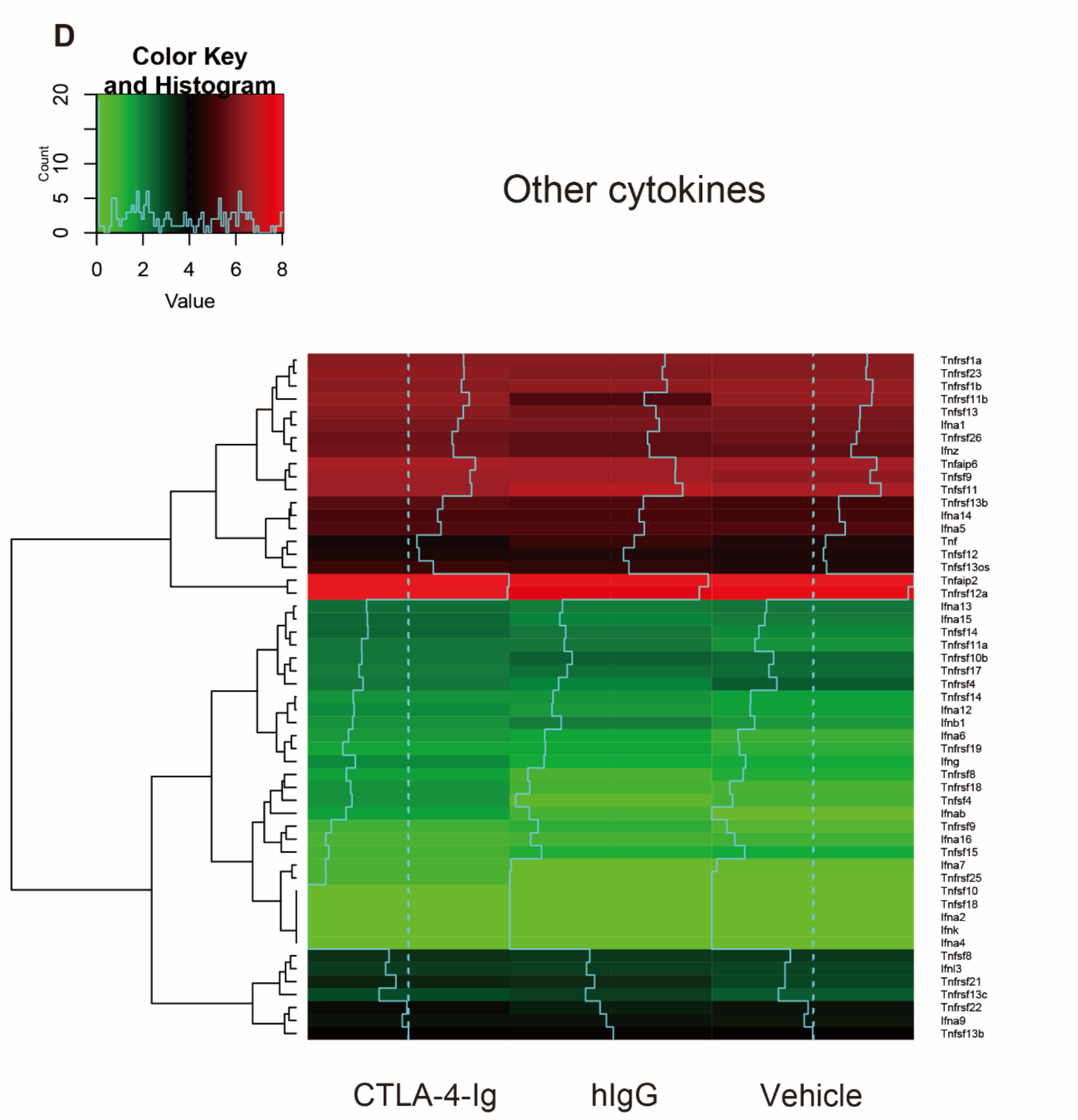

Supplementary figure 6D. No significant mRNA alternation associated with CTLA-4-Ig treatment in synovial cells

Microarray analysis of the isolated synovial cells treated with either CTLA-4-Ig, hIgG, or vehicle. Diagram showing heatmaps of representative other cytokines mRNAs.

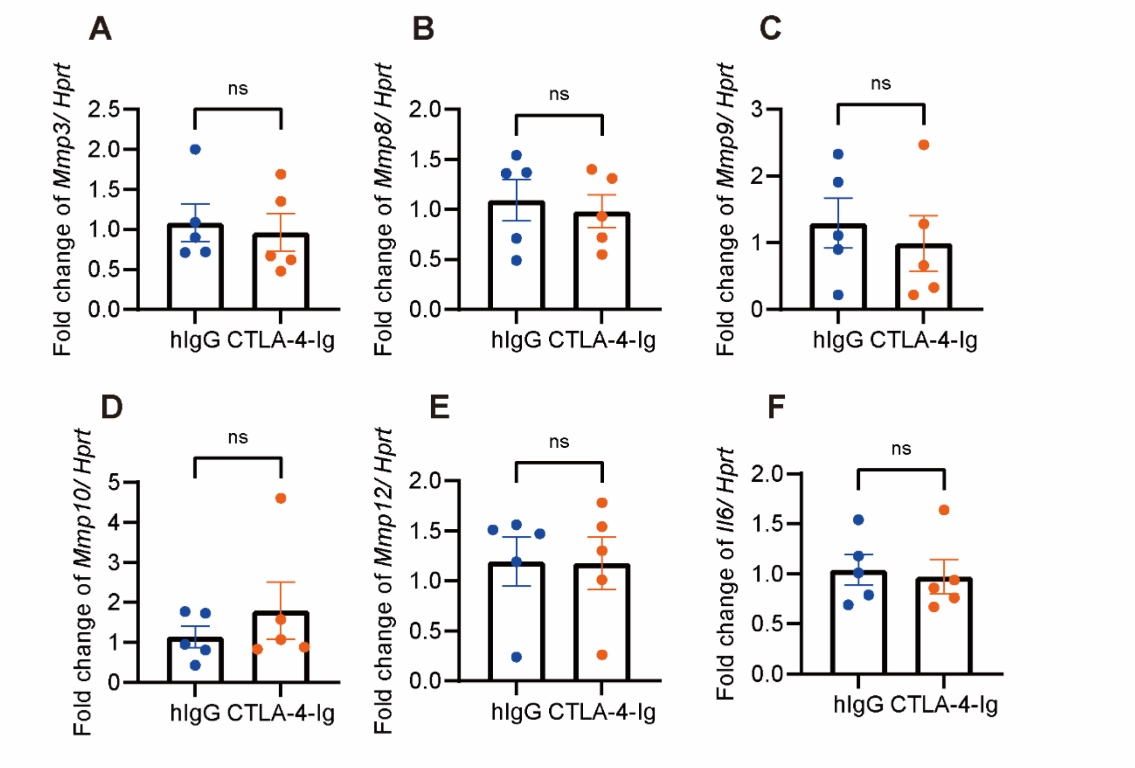

Supplementary figure 7. Significant alterations in various mRNA levels in synovial cells upon CTLA-4-Ig treatment were not observed when analyzed by qPCR. The expression of various genes in synovial cells treated with abatacept or hIgG was analyzed using qPCR. Fold changes in the expression levels of *Mmp3* (A), *Mmp8* (B), *Mmp9* (C), *Mmp10* (D), *Mmp12* (E), *Il6* (F). *Hprt* expression was used as an internal control for qPCR. Data are presented as the mean ± standard error (SE) of five mice.
